# eDNA Metabarcoding Reveals Homogenization of Fish in Fujiang Segments Isolated by Cascading Hydroelectric Stations

**DOI:** 10.3390/ani15142031

**Published:** 2025-07-10

**Authors:** Chao Deng, Shixia Huang, Bolin Chen, Rong Huang, Jiaqi Zhang, Zhihui Xiao, Chengcheng Ma, Zhijian Wang, Xiaohong Liu

**Affiliations:** 1 Key Laboratory of Freshwater Fish Reproduction and Development (Ministry of Education), Key Laboratory of Aquatic Science of Chongqing, School of Life Sciences, Southwest University Chongqing 400715, China; dc17749941501@163.com (C.D.);; 2 School of Agronomy, Xinjiang Hetian College, Hetian 848000, China

**Keywords:** cascade hydropower, eDNA, fish diversity, structural homogenization of fish communities

## Abstract

Background: The Fujiang River, a first-order branch of Jialing River, has for years been separated into six segments by six cascading hydropower stations in its downstream. However, the impact of cascading hydropower stations on its aquatic biota communities remains unclear. Methods: eDNA samples were collected in the upper, middle, and lower reaches of each river fragment during March, May, July, and December 2023, and after species identification, various statistical analyses including β-diversity, NMDS and MantelTest were performed using the R platform. Results: A total of 82 fish species belonging to 15 families were identified. The fish communities in the six fragments of the downstream Fujiang River showed a high degree of overlap, and a notable aggregation of fish communities between the upper, middle, and lower areas within each river section was also observed. Flow velocity (FV) and water temperature (TEMP) were found to be important factors in shaping fish distribution. Conclusion: Fish composition and distribution trend towards homogenization in the downstream of the Fujiang River.

## Simple Summary

The effect of cascade dams on freshwater ecosystems has received increasing attention recently. Using the eDNA technique, we found that in the downstream of the Fujiang River, which is divided into six fragments by five hydropower dams, the fish species’ richness, diversity, and distribution show homogenized trends. Contrary to the results of some previous studies, not water transparency, but water flow and temperature are driving factors in shaping fish diversity and distribution. This study provides a fundamental and more accurate basis for research on the distribution and evolution of fish community structure in cascading hydropower rivers.

## 1. Introduction

With the increasing emphasis on the protection of biodiversity, the effects of hydropower systems on river biosystems have received significant attention in the past few decades. In 2023, the global hydropower generation capacity reached 1397 GW, accounting for over 15% of global electricity production [1]. Unlike other power facilities, hydropower systems do not emit pollution or directly harm the environment with waste materials. Some reports have described hydropower systems as sustainable, eco-friendly, and clean energy sources which function by harnessing the natural flow of water [2]; however, their potential to cause ecological damage has been underestimated [3]. Recent studies have found that the operation of hydropower stations, especially cascading hydropower stations, can not only impact river flow conditions, river habitats, and riverbed substrates but can also alter river morphology [4,5] and therefore significantly affect riverine aquatic ecosystems.

Fish, as occupants of multiple trophic levels in aquatic ecosystems, play a crucial role in the stability of aquatic ecosystems [6]. Due to habitat fragmentation and changes in water flow characteristics (such as the duration and frequency of seasonal water levels), the presence of cascade dams can disrupt fish breeding habitats and negatively impact fish physiological activities and even survival [7,8]. Specifically, fish spawning activities have been reported to be altered after construction of the Three Gorges Dam in the Yangtze River [9]. By reducing river connectivity and thus blocking migration routes, the presence of dams has also been reported to hinder the exchange of fish populations between upstream and downstream areas and to make it difficult for migratory fish to reproduce [10,11,12]. Furthermore, recent studies on the cascading hydropower stations of the Jinsha River and Yongning River have discovered the negative impacts of hydropower stations on fish community structures, e.g., through severe miniaturization of river fish species, altered dominant species of fish, and altered distinct spatial distributions and community structural characteristics [13,14].

Current fish survey techniques mainly include conventional capture, newly developed environmental DNA (eDNA), and hydro-acoustic technology. In the context of China’s ten-year fishing ban policy in the Yangtze River, traditional fishery has been restricted, and eco-friendly monitoring technologies are widely used in recent and present fish monitoring. Acoustic technology is one of the widely applied techniques due to its ability to provide comprehensive, accurate, and timely information on fish resources and spatial locations in target areas [15,16,17]. However, it comes with an obvious limitation in species identification. eDNA technology is one of most popular and widely used non-invasive methods that has emerged in recent years. By sampling only water and then extracting, amplifying, and sequencing DNA and comparing biological information, this technology can provide information at both species and community structural levels, even elucidating the spatial distribution in the target area [18]. Now, it is widely applied in species detection in various categories including fish, zooplankton, phytoplankton, etc. all over the world [19,20,21], demonstrating particularly good reliability in fish-related resource monitoring and diversity assessments. For instance, using eDNA technology, 22 locally distributed fish species have been identified in the Beagle Channel (which is in accordance with historical records [22], demonstrating the high accuracy of eDNA technology). Moreover, by using eDNA, significant differences in the diversity and community stability of fish between the mainstem and the tributaries of the Rui River have been found, and a previously unknown endemic fish genus *Triplophysa*, with high diversity, has contributed significantly to community stability [23]. Thus, the non-invasive and environmentally friendly characteristics of eDNA technology perfectly meet the requirements of current ecological conservation.

The Fujiang River is a secondary tributary of the Yangtze River and the largest right-bank branch of the Jialing River. Since the 1970s, a total of six cascading hydropower stations have been successively constructed in the lower reaches of the Fujiang River, dividing the 129 km long downstream of this river (from Sanxing Power Station in Suining to the confluence with the Jialing River) into six fragments, with approximately 20 km in each section; this river therefore represents an ideal model for investigating the ecological impacts of cascading hydropower stations on river segments and freshwater aquatic organisms.

In order to clarify the fish community structure and spatial–temporal distribution characteristics in the river with continuous cascading hydropower stations and thus to investigate the effects of cascade dams on fish communities, eDNA samples and corresponding environmental factors from these six river fragments downstream of the Fujiang River were collected in March, May, July, and December 2023. Then the differences in fish community structure among the various sampling sites and their relationships with environmental factors were analyzed. The results herein can provide basic data information for the protection of ecological diversity in the Yangtze River Basin, and they will benefit further exploration of the impacts of cascading hydropower stations on the spatial–temporal distribution characteristics of fish in freshwater river basins.

## 2. Materials and Methods

### 2.1. Research Area

**Figure 1:**
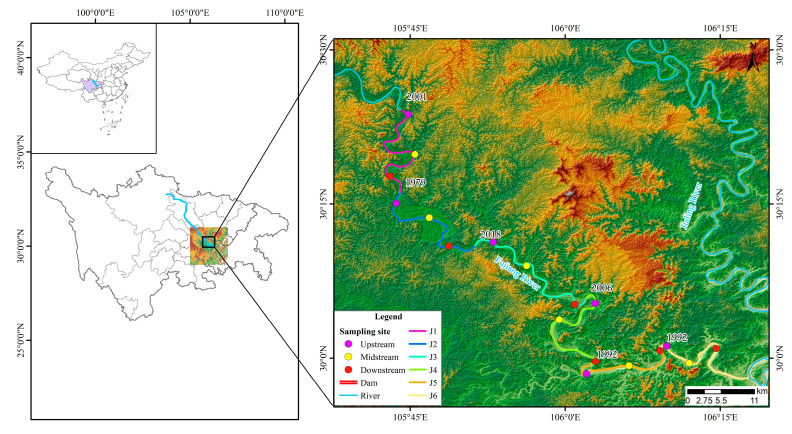
Sampling sites. The numbers beside the dams indicate the years of dam construction. Sanxing: Sanxing Hydropower Station; Sankuaishi: Sankuaishi Hydropower Station; Tongnan: Tongnan Navigation Hub; Fujin: Fujin Hydropower Station; Anju: Anju Hydropower Station; Weituo: Weituo Hydropower Station. J1: segment from Sanxing Hydropower Station to Sankuaishi Hydropower Station; J2: segment from Sankuaishi Hydropower Station to Tongnan Navigation Hub; J3: segment from Tongnan Navigation Hub to Fujin Hydropower Station; J4: segment from Fujin Hydropower Station to Anju Hydropower Station; J5: segment from Anju Hydropower Station to Weituo Hydropower Station; J6: segment from Weituo Hydropower Station to confluence with the Jialing River.

The study area of this paper extends from the Sanxing Hydropower Station to the confluence with the Jialing River in the downstream of the Fujiang River (E 105°43′, E 106°16′; N 30°24′, N 29°59′). There are six hydropower stations dividing the river into six independent river segments, namely, J1—the segment from Sanxing Hydropower Station to Sankuaishi Hydropower Station; J2—the segment from Sankuaishi Hydropower Station to Tongnan Navigation Hub; J3—the segment from Tongnan Navigation Hub to Fujin Hydropower Station; J4—the segment from Fujin Hydropower Station to Anju Hydropower Station; J5—the segment from Anju Hydropower Station to Weituo Hydropower Station; and J6—the segment from Weituo Hydropower Station to confluence with the Jialing River (Figure 1). All six of these hydropower stations were constructed from 1992 to 2002 (Table 1).

### 2.2. eDNA Sampling

eDNA samples were collected in March, May, July, and December 2023, and a total of 48 samples were collected for each river segment. Samples were collected at 3 sites (Table S1)—upstream, midstream, and downstream—and 4 samples (including 3 technical replicates and 1 negative control) were collected at each site. The upstream sampling site was designed to be within 3 km of the dam, representing the dam tailwater lotic habitat. The midstream point was situated near the middle of the river fragment, representing the moderate-flow habitat. The downstream point was positioned within 3 km above the next dam, representing the reservoir lentic habitat. Notably, the downstream sampling point of J6 was moved forward to avoid the influence of fish from the Jialing River. During the sampling process, 1 L of water was collected below the water surface at 0.5 m via a water sampler and subsequently filtered through a 0.45 μm mixed cellulose filter membrane (Whatman, Maidstone, UK) utilizing a portable eDNA sampling device (SMITH-ROOT, Vancouver, WA, USA). Before sampling at each site, all related equipment and containers were disinfected with a 10% bleach solution to avoid contamination, and 1 L of pure water served as a negative control. After filtration, all filter membranes were stored at −20 °C until use.

### 2.3. Environmental Factor Measurement

Various environmental factors including water temperature (TEMP), dissolved oxygen (DO), pH value, flow velocity (FV), salinity (SAL), electrical conductivity (EC), and transparency of each sampling site were measured and recorded by the thermometer (AR827, SMART SENSOR, Dongguan, China), portable dissolved oxygen meter (AR8406, SMART SENSOR, Shenzhen, China), pen-type pH meter (PH828, SMART SENSOR, Shenzhen, China), portable flow meter (LS300-A, JINSHUI, Zhengzhou, China), pen-type salinity meter (AR8212, SMART SENSOR, Shenzhen, China), conductivity meter (AR8011, SMART SENSOR, Shenzhen, China).

### 2.4. Database Construction

A local database of fish species was constructed based on research related to fish resources surveys in the middle and upper reaches of the Yangtze River and the Fujiang River over the years, alongside published books including “Fish of the Yangtze River” [24], “Fish of Sichuan” [25], and “Color Atlas of Fishes in Sichuan” [26]. All freshwater fish 12SrRNA and mitochondrial genome sequences from the nucleotide database on NCBI were retrieved (National Center for Biotechnology Information, https://www.ncbi.nlm.nih.gov, (accessed on 10 November 2022)) to serve as a reference database for eDNA annotation.

### 2.5. eDNA Extraction and Sequencing

DNA was extracted from the mixed cellulose filter membrane using a PowerWater DNA Isolation Kit, and sample quality was assessed using 1% agarose gel electrophoresis. PCR amplification was performed using tele02 primers (tele02-F: 5′-AAA CTC GTG CCA GCC ACC-3′; tele02-R: 3′-GGG TAT CTA ATC CCA GTT TG-5′) [27], with PCR conditions consistent with a previous report [28]. No amplification was observed in the negative control group. Agarose gel electrophoresis was used to confirm the singularity and specificity of the amplification products, and PCR products were purified using an equal volume of Agencourt AMPure XP nucleic acid purification beads (Beckman coulter, Brea, CA, USA). Sequencing of the libraries was performed on the NovaSeq 6000 platform (Illumina, San Diego, CA, USA) using the SP-Xp (PE250) paired-end sequencing strategy, followed by bioinformatics analysis.

### 2.6. Bioinformatics

The raw read sequences were processed in QIIME2 [29]. The adaptor and primer sequences were trimmed using the cutadapt plugin. The DADA2 plugin was utilized for quality control and amplicon sequence variant (ASV) identification [30]. Taxonomic assignments of ASV representative sequences were carried out with a confidence threshold of 0.8 using a pre-trained Naïve Bayes classifier trained on 12S (version 2023_02_07). Once two or more sequences from public database showed the same or similar correspondence with an ASV, the taxonomic assignment processes complied with the following criteria to improve accuracy: (1) priority to endemic species in local databases (e.g., endemic to Sichuan, the Yangtze River, and Chinese); (2) secondary matching to georeferenced NCBI sequences and exclusion of extra-regional distributed species; and (3) downgrading the identification to genus/family level.

### 2.7. Diversity Analysis

The results of eDNA fish presence at each sampling site were analyzed and visualized using R and the community ecology package [31] for univariate analysis and plotting. Beta diversity was utilized to analyze differences in community structure among different regions within and between river sections. The R packages ade4, scatterplot3d, and phyloseq (v1.7.13, v0.3.41, v1.26.1) were employed to calculate species’ Bray–Curtis distances and generate NMDS (non-metric multidimensional scaling) plots for upstream, midstream, and downstream of each fragment within and between river segments based on Bray–Curtis distances. Statistical significance was assessed using the ADONIS test, with significance set at *p* < 0.05.

### 2.8. Correlation Analysis of Environmental Factors

MantelTest analysis was performed on the OTU matrix and environmental factor matrix using the R package vegan (v2.5-7). Based on Spearman correlation analysis, we assessed the correlation between species and environmental factors. An R package heatmap (v1.0.12) was used for visualization. Detrended correspondence analysis (DCA) was conducted on species composition data, resulting in a first axis length value of 2.11, which was less than 3.0. Therefore, redundancy analysis (RDA) was chosen to examine the relationship between fish communities and environmental factors. All collected environmental factors were analyzed using the vegan package in R (v2.5.6) and visualized using the ggplot2 package (v3.3.0).

## 3. Results

### 3.1. Diversity of Fish Species in Different River Segments

**Figure 2:**
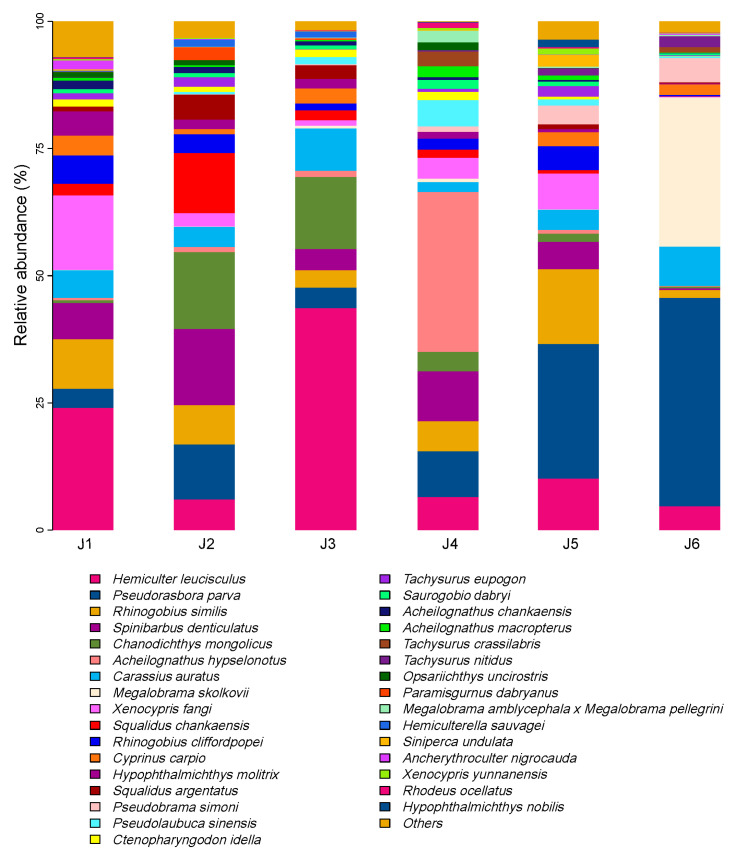
Species composition of fish based on relative sequence abundance of each river section. The species with an OTU abundance exceeding 1% are displayed. J1: segment from Sanxing Hydropower Station to Sankuaishi Hydropower Station; J2: segment from Sankuaishi Hydropower Station to Tongnan Navigation Hub; J3: segment from Tongnan Navigation Hub to Fujin Hydropower Station; J4: segment from Fujin Hydropower Station to Anju Hydropower Station; J5: segment from Anju Hydropower Station to Weituo Hydropower Station; J6: segment from Weituo Hydropower Station to confluence with the Jialing River.

**Figure 3:**
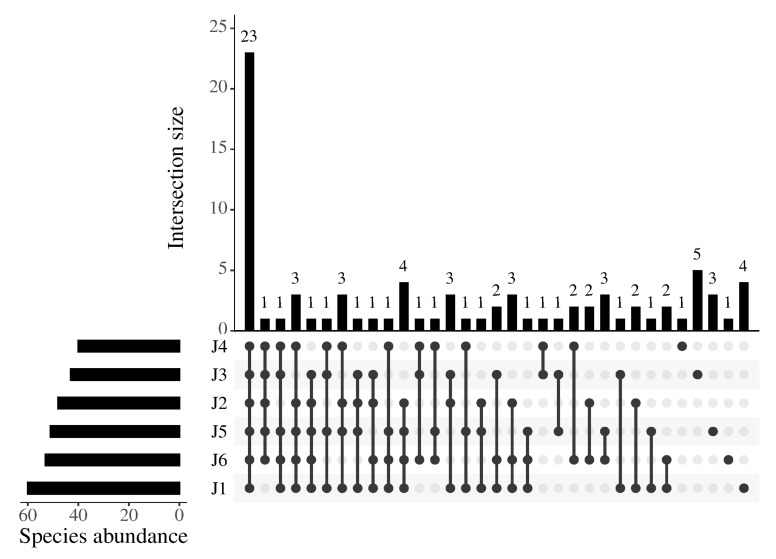
Upset diagram of species composition in six river fragments of the downstream Fujiang River. J1: segment from Sanxing Hydropower Station to Sankuai‘shi Hydropower Station; J2: segment from Sankuaishi Hydropower Station to Tongnan Navigation Hub; J3: segment from Tongnan Navigation Hub to Fujin Hydropower Station; J4: segment from Fujin Hydropower Station to Anju Hydropower Station; J5: segment from Anju Hydropower Station to Weituo Hydropower Station; J6: segment from Weituo Hydropower Station to confluence with the Jialing River.

In this study, a total of 645 valid ASVs were obtained, belonging to 15 families and 82 fish species (Table S2). Among them, the Cyprinidae family comprised 51 species, representing the largest proportion (62.20%). The top five fish species with the highest relative abundance of OTUs were *Hemiculter leucisculus*, *Pseudorasbora parva*, *Rhinogobius similis*, *Spinibarbus denticulatus*, and *Chanodichthys mongolicus* (Figure 2). A total of 23 fish species were found in the 6 river segments, predominantly consisting of Cyprinidae species such as *Hemiculter leucisculus* and *Xenocypris fangi* (Figure 3).

### 3.2. Composition of Fish Communities in Continuous River Segments

**Figure 4:**
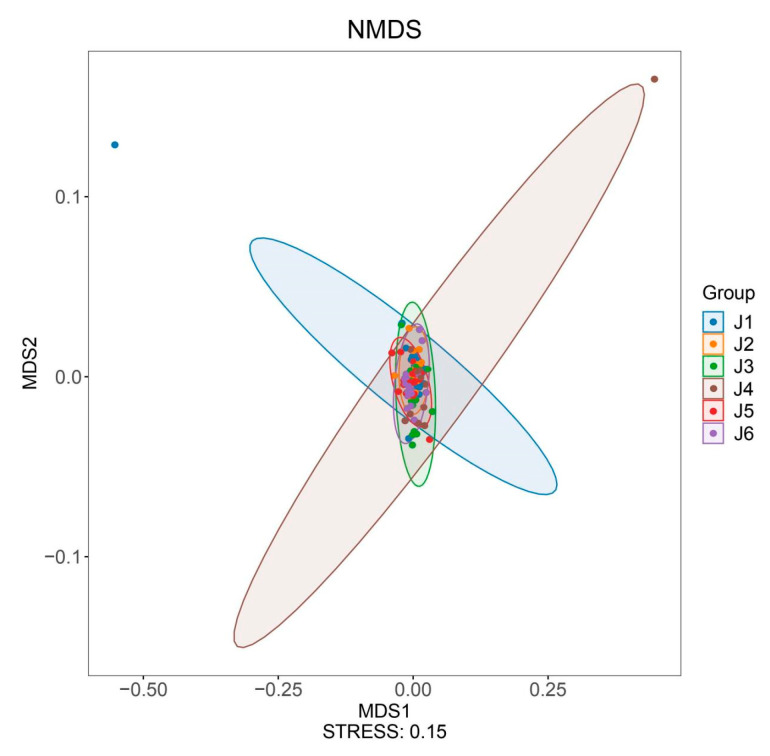
NMDS analysis plot of fish composition for six river sections. J1: segment from Sanxing Hydropower Station to Sankuaishi Hydropower Station; J2: segment from Sankuaishi Hydropower Station to Tongnan Navigation Hub; J3: segment from Tongnan Navigation Hub to Fujin Hydropower Station; J4: segment from Fujin Hydropower Station to Anju Hydropower Station; J5: segment from Anju Hydropower Station to Weituo Hydropower Station; J6: segment from Weituo Hydropower Station to confluence with the Jialing River.

Hydropower stations divide continuous river segments into multiple relatively independent sections, resulting in a certain degree of geographic isolation. Beta diversity can reflect the differences in community structure and species composition between different geographical locations. Using NMDS for beta diversity analysis of the six river segments, the clustering results show a high level of aggregation among the segments (stress = 0.15, Figure 4), indicating similar fish community structures among these six river fragments (based on Bray–Curtis distance, ADONIS test, R^2^ = 0.099, *p* = 0.001).

### 3.3. Composition of Fish Communities Within River Segments

**Figure 5:**
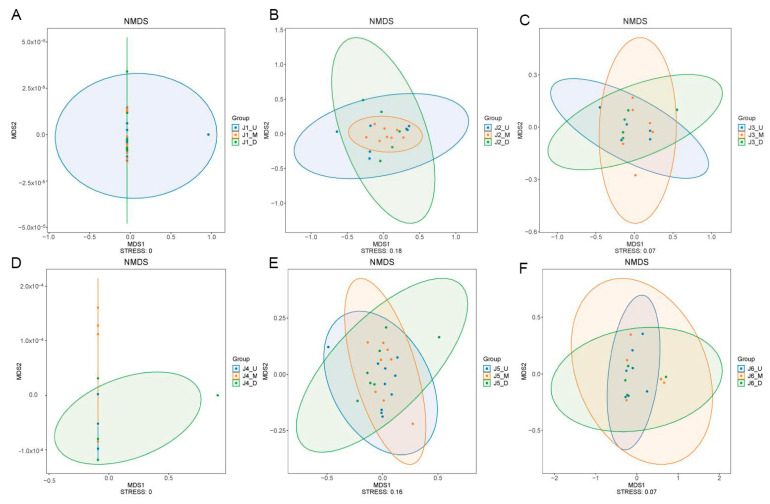
NMDS analysis plots of fish composition in the upstream, midstream and downstream of each river section. (**A**): NMDS results for J1; (**B**): NMDS results for J2; (**C**): NMDS results for J3; (**D**): NMDS results for J4; (**E**): NMDS results for J5; (**F**): NMDS results for J6. J1: segment from Sanxing Hydropower Station to Sankuaishi Hydropower Station; J2: segment from Sankuaishi Hydropower Station to Tongnan Navigation Hub; J3: segment from Tongnan Navigation Hub to Fujin Hydropower Station; J4: segment from Fujin Hydropower Station to Anju Hydropower Station; J5: segment from Anju Hydropower Station to Weituo Hydropower Station; J6: segment from Weituo Hydropower Station to confluence with the Jialing River.

In this study, NMDS was used to conduct beta diversity analysis on the fish composition of the upstream, midstream, and downstream sections of all six river segments. The stress values of NMDS analysis for the upstream, midstream, and downstream sections of the six river segments are all below 0.2 (J1: stress = 0, J2: stress = 0.18, J3: stress = 0.07, J4: stress = 0, J5: stress = 0.16, J6: stress = 0.07, Figure 5), showing a good reflection of distances between samples. The separate analytical result charts for the six river segments all demonstrate a high degree of overlap in the fish community structures within their respective upper, middle, and lower reaches. We then conducted an ADONIS test on all the NMDS analytical results. The test results for J1 and J5 are significant (ADONIS test, J1, R^2^ = 0.1211, *p* = 0.0228; J5, R^2^ = 0.1215, *p* = 0.0319), while the results for J2, J3, J4, and J6 are not significant (ADONIS test, J2, R^2^ = 0.1285, *p* = 0.1436; J3, R^2^ = 0.1366, *p* = 0.3938; J4, R^2^ = 0.1705, *p* = 0.8174; J6, R^2^ = 0.1344, *p* = 0.4283).

### 3.4. Impact of Environmental Factors on Fish Species

**Figure 6:**
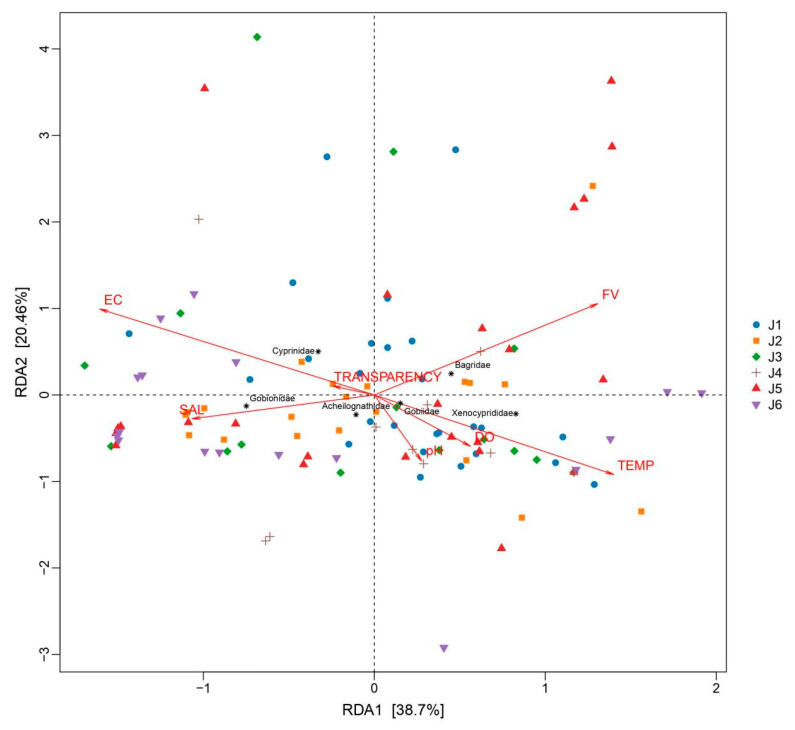
RDA plot of environmental factors and fish species (at the family level) in the six river segments. J1: segment from Sanxing Hydropower Station to Sankuaishi Hydropower Station; J2: segment from Sankuaishi Hydropower Station to Tongnan Navigation Hub; J3: segment from Tongnan Navigation Hub to Fujin Hydropower Station; J4: segment from Fujin Hydropower Station to Anju Hydropower Station; J5: segment from Anju Hydropower Station to Weituo Hydropower Station; J6: segment from Weituo Hydropower Station to confluence with the Jialing River. DO: dissolved oxygen; FV: flow velocity; TEMP: water temperature; EC: electrical conductivity; SAL: salinity.

**Figure 7:**
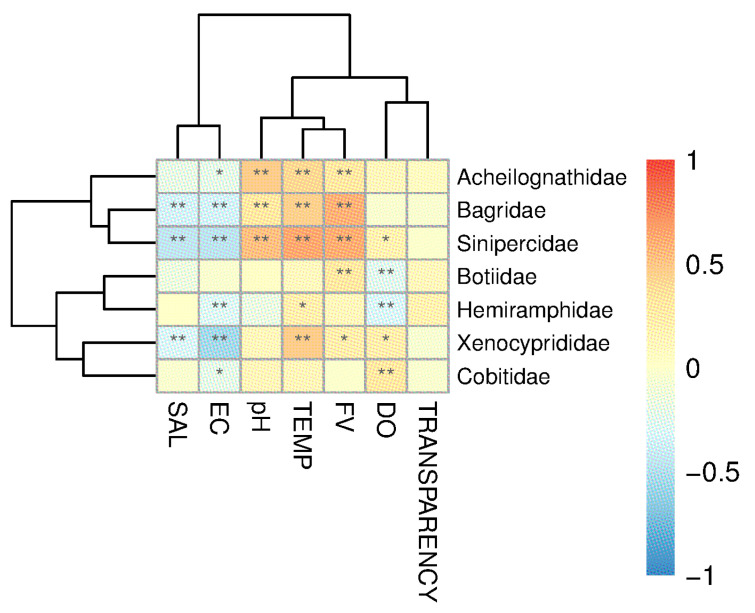
Correlation between fish species (at the family level) and environmental factors (correlation coefficients > 0.3, *: *p* < 0.05, **: *p* < 0.01). DO: dissolved oxygen; FV: flow velocity; TEMP: water temperature; EC: electrical conductivity; SAL: salinity.

A MantelTest was conducted to test the correlation between the OTU and the environmental factors (Table S3), revealing a significant relationship between environmental factors and fish distribution (MantelTest, r = 0.2153, *p* = 0.0001). The RDA analysis results (Figure 6) showed that the explanatory power of the first axis is 38.7%, and the second axis is 20.46%. Electrical conductivity, flow rate, and water temperature are the top three environmental factors that have the greatest impact on fish distribution (Figure 6). A further Spearman correlation analysis between environmental factors and several main fish categories showed that transparency shows no significant correlation with fish composition (Figure 7); the distribution of Bagridae and Xenocyprididae fish species is significantly positively correlated with FV and TEMP (*p* < 0.05), while the distribution of Gobiidae and Gobionidae fish species shows weak correlation with the environmental factors (Figure 7). Detailed environmental factor data are provided in Table S3.

## 4. Discussion

### 4.1. Similar Fish Community Structures Among Continuous Cascading Hydropower Station River Segments

As illustrated above, the extremely high degree of overlap in the fish community structures in the research area in this study indicates that, within 120 km, the fish community structures in continuous cascading hydropower station river segments tend to be consistent.

Construction of cascading hydroelectric stations can lead to a decrease in the diversity of fish species in rivers [32,33]. However, with the continuous operation of dams, river habitats can be gradually stabilized, leading to a recovery and an increase in fish diversity [34], ultimately resulting in a phenomenon of community structure homogenization [28]. In the present study, the NMDS results for the six segments showed a high degree of aggregation in fish community structure, indicating that the fish community compositions in the six segments are similar, consistent with the research findings in the Wujiang River (which also features several cascaded hydropower stations) [28]. This effect may be attributed to the construction of multiple dams over 20 years ago (Figure 1), leading to the formation of homogenized fish communities over the past few decades [28,35]. Additionally, the original connection between the various river segments resulted in similar fish community structures; in comparison with natural river habitat changes, the impact of dams and reservoirs on fish communities is less significant [36], meaning there is a relatively stable and similar community structure even after significant changes.

### 4.2. Uniformity in Fish Community Structures Within One Independent River Segment Isolated by Cascading Hydropower Stations

Previous studies have often focused on differences in fish diversity among river segments isolated by cascading hydropower stations; however, the potential differences in fish community structures among the upstream, midstream, and downstream sections within one individual river segments have always been overlooked [10,12]. In the present study, the results of NMDS analysis of fish community composition for each river section (Figure 5) suggested a similar fish species composition in the upper, middle, and downstream of each fragment in the 20 km long river section, which further indicates a trend towards uniformity in fish community structures within each river segment.

The distance between cascading hydroelectric stations and their operational intensity can influence the fish community structure; specifically, fish that are more adaptable to the environment may be less impacted by the closer proximity and more intensive operation of the stations [37]. Studies have focused on comparing fish diversity between the upstream reservoir areas and downstream flowing water areas of individual dams, typically showing differences in fish composition between the reservoir storage area and the downstream flowing river segment [13,37,38]. For small hydroelectric watersheds, changes in fish community composition in rivers under the influence of dams tend to develop towards a structure associated with stagnant water [39]. The focus of this study was river segments with a length of around 20 km (divided by cascading hydroelectric stations), and habitat differences at the 20 km scale may not be significant [5,40]. This has resulted in strong consistency in fish community structures across the various river segments, where habitats might have stabilized after the long-term operation of the stations. A previous study showed that sand mining operations can impact aquatic ecosystems and alter fish community structures [41,42]. During the sampling periods, sand mining was also observed in some river segments in the study area. The NMDS statistical results above show a similar fish community structure in the upper, middle, and lower reaches of the respective river sections of all six segments, and the ADONIS test results of J1 and J5 were significant. The remaining four segments showed uniformity in fish composition, but the test results were not significant. We inferred that the sand mining activities and their frequency in these river segments were also factors affecting fish composition. Certainly, there are other factors influencing the fish community structure, and these await further research.

### 4.3. Impact of Environmental Factors on Fish Distribution

Available reports have shown that in natural river basins, factors such as altitude and riverbed substrate can influence fish distribution [43]. Water salinity, temperature, conductivity, and flow rate also affect fish distribution [44,45,46], fish reproductive cycles, and other life cycles [18,47]. However, in rivers where there is human interference, such as the presence of dams, the situation changes. On one hand, as the habitat stabilizes, the originally flowing river gradually becomes homogenous, showing less variation [28]. On the other hand, the fish species found in rivers with cascade hydropower stations are mostly those that are adaptable and insensitive to environmental factors [48,49]. Cascade dams can also lead to an overall increase in water transparency in river bodies [50], and the transparency levels sampled in the river segments in this study were all at a similarly high level (above 40 cm), showing a relatively balanced basin-wide transparency and resulting in minimal impact on the fish community’s composition. With the operation of dams, the flood discharge and water storage inevitably change the water temperature and flow velocity, which in turn create distinct microhabitats in the river; there are lotic conditions downstream and lentic conditions upstream of dams. Consequently, flow velocity emerges as a critical determinant of the composition of the fish community. This phenomenon becomes particularly pronounced during the high-flow season (Table S3). Simultaneously, thermal gradients induced by flow velocity variations also render water temperature a significant contributor to differentiation of the fish community [51]. It is worth noting that, based on the RDA results, the distribution of Gobiidae and Gobionidae fish species shows a weak correlation with environmental factors in river sections isolated by cascading hydropower stations. These fish are small in body length, and even mature individuals are less than 10 cm. Fish with a small body length are reported to be more adaptive and resistant to complex and variable environments [52]; thus, fish species with body lengths like Gobiidae and Gobionidae fish are better able to survive in rivers with cascaded hydropower stations and maintain a relatively dominant position in terms of OTUs. Compared to historically documented fish species in the Fujiang River, several endemic rheophilic species and drifting egg-laying species—such as *Leptobotia elongata*—were not recorded in the current survey [53]. Endemic fish species in rivers often rely on specific habitats or environmental factors for survival, and their presence is crucial for maintaining the stability of community structures [23]. The existence of cascading hydropower stations tends to homogenize environmental factors such as flow velocity and water temperature across river sections (Table S2), which inevitably disrupts unique habitats and causes the local extinction of endemic species.

### 4.4. The Limitations of eDNA Technology

The emergence and development of environmental DNA (eDNA) methods have significantly advanced ecological research, yet current limitations persist [54]. For instance, when fish eDNA is detected in reservoir areas upstream of dams, it remains uncertain whether it can be transported via flowing water to downstream locations for repeated detection. Moreover, eDNA signals may originate from effluence of high-density aquaculture operations near rivers rather than from natural fish populations. The available research has pointed out that fish eDNA typically remains detectable within approximately 2 km downstream [55]. Although our sampling design maximized site representativeness and independence by spacing locations > 3 km apart, the possibility of interference cannot be completely eliminated. Furthermore, eDNA results depend critically on the completeness of the reference species database and the accuracy of sequence matches. Given the prevalence of misannotated records in public repositories, we systematically cross-verified computational classifications against historically documented checklists of local species, thereby providing evidence for the presence of identified fish in the study area.

## 5. Conclusions

Although previous research has indicated that the fish community structures in cascading hydropower station river segments may exhibit a trend towards homogeneity [28], in the present study, we selected more regular and rigorous research areas and confirmed the accuracy and rationality of this trend. Furthermore, this study analyzed whether there is differentiation in fish community structure at different locations within a specific cascading hydropower station river segment. The results show that the entire river section’s fish community has a tendency to develop towards the same community structure.

The presence of dams drives the homogenization of fish assemblages and environmental factors, resulting in the loss of ecosystem diversity. This process may further lead to the extirpation of endemic species. Natural rivers must be protected from the impacts of damming, and inappropriately sited dams should be decommissioned progressively.

## Figures and Tables

**Table 1:** Basic information of the hydropower facilities in the lower reaches of the Fujiang River.

No.	Hydropower Facility Name	Storage Level (m)	Dam Type	Completion Time	Fish Passage Facilities
1	Sanxing	262.5	High Dam	2001	No
2	Sankaishi	246.0	Low Dam	1979	No
3	Tongnan	236.0	High Dam	2018	No
4	Fujin	229.0	High Dam	2006	No
5	Anju	216.0	High Dam	1992	No
6	Weituo	206.0	High Dam	1992	No

## Data Availability

Data will be made available on request.

## Supplementary Materials

The following supporting information can be downloaded at https://www.mdpi.com/article/10.3390/ani15142031/s1, Table S1: Information of the all sampling sites. Table S2: Fish species composition and proportional abundance monitored by eDNA. Table S3: Environmental factors at sampling sites.

## Abbreviations

The following abbreviations are used in this manuscript:

eDNA	Environmental deoxyribonucleic acid
OTUs	Operational taxonomic units
RDA	Redundancy analysis
FV	Flow velocity
TEMP	Water temperature
DO	Dissolved oxygen
SAL	Salinity
EC	Electrical conductivity
PCR	Polymerase chain reaction
ASVs	Amplicon sequence variants
NMDS	Non-metric multidimensional scaling
DCA	Detrended correspondence analysis
