# Supplementary material for: eDNA Metabarcoding Reveals Homogenization of Fish in Fujiang Segments Isolated by Cascading Hydroelectric Stations
Source: Animals (Basel). 2025 Jul 10;15(14):2031. doi: 10.3390/ani15142031 (PMC12291789; doi:10.3390/ani15142031)
Supplement: Supplementary file 1 [file animals-15-02031-s001.zip › animals-3717731-supplementary.pdf]

Table S1 Information of the all sampling sites

No.	Segment	Site	Longitude	Latitude	Altitude
1	J1: Sanxing Hydropower Station to Sankuaishi Hydropower Station	J1-U	105.7657397	30.38108026	254.968
2		J1-M	105.7758248	30.32129918	246.469
3		J1-D	105.7384885	30.28903757	245.6
4	J2: Sankuaishi Hydropower Station to Tongnan Navigation Hub	J2-U	105.7481444	30.24845575	242.409
5		J2-M	105.7969606	30.22710537	235.871
6		J2-D	105.8261001	30.18506979	233.097
7	J3: Tongnan Navigation Hub to Fujin Hydropower Station	J3-U	105.8918035	30.19071316	229.451
8		J3-M	105.9410703	30.15556549	227.016
9		J3-D	106.0127926	30.09805893	224.674
10	J4: Fujin Hydropower Station to Anju Hydropower Station	J4-U	106.0428333	30.0997219	220.303
11		J4-M	105.989275	30.07551764	217.122
12		J4-D	106.0431338	30.01320456	216.532
13	J5: Anju Hydropower Station to Weituo Hydropower Station	J5-U	106.0297871	29.99485825	211.4
14		J5-M	106.1226988	29.99994372	205.532
15		J5-D	106.1384916	30.02936219	204.92
16	J6: Weituo Hydropower Station to Confluence with the Jialing River	J6-U	106.1484909	30.03642176	203.96
17		J6-M	106.1818737	30.01054649	202.459
18		J6-D	106.221506	30.03252988	201.058

Table S2 Fish species composition and abundance proportion monitored by eDNA

Family	Species	J1	J2	J3	J4	J5	J6
Acipenseridae	*Acipenser sp. x Acipenser sp.*	--	+	--	--	--	+
Cyprinidae	*Carassius auratus*	+++++	+++++	+++++	++	+++++	+++++
	Cyprinus carpio	++++	++++	++++	--	++++	++++
	Ctenopharyngodon idella	++++	++++	++++	++++	++++	++
	Hypophthalmichthys molitrix	++++	++++	++++	++++	++++	+++
	Hypophthalmichthys nobilis	++	++	--	+++	++++	--
	Hemiculter leucisculus	+++++	+++++	+++++	++++	+++++	++++
	Squaliobarbus curriculus	+++	--	--	--	--	+++
	Pseudorasbora parva	++++	+++++	++++	+++++	+++++	+++++
	Saurogobio dabryi	++++	++++	+++	--	--	--
	Hemibarbus maculatus	+++	--	++	--	--	--
	Opsariichthys uncirostris	++++	++++	--	++++	+++	--
	Zacco platypus	++	+++	+++	--	--	--
	Pseudobrama simoni	++	+	++	+++	+++++	+++++
	Pseudolaubuca sinensis	+	+++	++++	++++	++++	+++
	Cultrichthys erythropterus	+++	+++	--	--	--	++
	Culter alburnus	+++	+++	+	+	+++	+++
	Culter mongolicus	+++	+++++	+++++	+++++	++++	++
	Ancherythroculter nigrocauda	++++	+++	--	--	+++	+++
	Ancherythroculter wangi	++	--	--	--	--	--
	Megalobrama pellegrini	+++	++	+++	+++	+++	+++++
	Megalobrama skolkovii	--	--	--	--	+	+
	Mylopharyngodon piceus	+++	--	--	--	+++	+++
	Cirrhinus mrigala	--	--	++	--	--	--
	Abbottina obtusirostris	++	--	+++	--	--	++

	Gnathopogon herzensteini	+++	--	--	--	--	--
	Microphysogobio tungtingensis	--	--	--	--	+++	+++
	Squalidus wolterstorffi	++	+++++	++++	++++	++++	++
	Squalidus argentatus	++++	++++	++++	++	++++	+++
	Sarcocheilichthys davidi	+++	--	+	+	+++	--
	Hemiculterella sauvagei	+++	++++	++++	+	+	+
	Sinibrama taeniatus	+++	++	--	--	+++	++
	Rhodeus ocellatus	+++	--	+++	+++	+++	+++
	Rhodeus sinensis	--	--	--	--	++	--
	Acheilognathus hypselonotus	+++	++++	++++	+++++	++++	+++
	Acheilognathus chankaensis	++++	++++	+++	+++	+++	+++
	Acheilognathus macropterus	+++	+++	+++	++++	++++	+++
	Spinibarbus denticulatus	+++	+++	--	+++	+++	+++
	Spinibarbus sinensis	+++++	+++++	+++	+++++	+++++	+++
	Xenocypris fangi	+++++	++++	+++	++++	+++++	+++
	Xenocypris davidi	+++	+++	--	+++	++++	+++
	Distoechodon tumirostris	++	+++	--	--	++++	+++
	Acrossocheilus monticola	+++	--	--	--	--	--
	Onychostoma alticorpus	--	--	--	+	--	--
	Abbottina rivularis	--	--	+	+	--	--
	Hemiculter tchangi	+	--	+	--	--	+
	Hemibarbus labeo	+	+	+	--	--	+
	Gnathopogon imberbis	+	+	--	+	+	+
	Acheilognathus typus	+	+	--	--	+	+
	Microphysogobio fukiensis	+	+	--	--	--	+
Cobitidae	*Misgurnus anguillicaudatus*	++	--	--	--	--	--
	Paramisgurnus dabryanus	+++	++++	+++	--	--	--
Botiidae	*Parabotia bimaculata*	--	--	--	--	+++	--

	Sinibotia superciliaris	--	--	--	--	+++	--
	Parabotia fasciatus	+	+	--	--	+	--
Balitoridae	*Sinogastromyzon szechuanensis*	--	--	+	--	+++	--
	Lepturichthys fimbriata	+	--	--	--	--	+
Gobiidae	*Rhinogobius giurinus*	+++++	+++++	++++	++++	+++++	++++
	Rhinogobius cliffordpopei	++++	++++	++++	++++	+++++	+++
	Rhinogobius brunneus	+++	+++	+	--	+++	--
	Micropercops swinhonis	--	--	++	--	--	--
	Rhinogobius similis	+	+	--	--	--	+
	Mugilogobius myxodermus	--	--	--	+	++++	+++
Bagridae	*Pelteobagrus eupogon*	++++	++++	+	+++	++++	++
	Pelteobagrus fulvidraco	+++	++	--	--	--	--
	Pelteobagrus nitidus	++	++	++	+++	++++	++++
Synbranchidae	*Monopterus albus*	--	--	+	--	--	--
Hemiramphidae	*Hyporhamphus intermedius*	--	--	--	--	+	++
Channidae	*Channa argus*	++	--	--	+	+++	++
	Channa gachua	--	--	--	--	--	+
Clariidae	*Clarias batrachus*	--	--	+	+	--	--
Poeciliidae	*Gambusia affinis*	--	++	--	--	--	++
Cichlidae	*Oreochromis niloticus*	+++	+	--	+	+	--
Siluridae	*Silurus asotus*	++	--	--	+	+++	--
	Silurus meridionalis	+++	+++	--	--	--	--
Percichthyidae	*Siniperca chuatsi*	+++	--	--	--	+++	--
	Siniperca kneri	+++	++	++	+++	++++	+
	Siniperca obscura	--	--	--	++	--	+
Hybrids	*Tachysurus sp.× Tachysurus sp.*	--	--	+	--	--	--
	Megalobrama sp.× Megalobrama sp.	--	+++	++++	++++	++++	+++++
	Ctenopharyngodon idellus × Elopichthys bambusa	--	--	--	+	--	++

Cyprinus carpio × *Megalobrama amblycephala*	--	--	+	+	--	+

Note: Symbol definitions: "+++++" denotes >1% relative biomass proportion; "++++" = 0.1–1%; "+++" = 0.01–0.1%; "++" = 0.001–0.01%; "+" = 0.0001–0.001%; "--" indicates undetected species at sampling sites, with scientific nomenclature following *Fauna Sinica: Osteichthyes*.

Table S3 Environmental factors at sampling sites

Time	Site	TEMP （℃）	DO （mg/L）	pH	FV （M/s）	SAL	EC （μs/cm）	TRANSPARENCY （cm）
March	J1-U	16.7	10.34	8.57	0.05	0.17	530	70
	J1-M	17.6	9.23	8.56	0.02	0.18	514	50
	J1-D	19.4	10.28	8.45	0.02	0.18	532	40
	J2-U	17.8	9.56	8.72	0.01	0.19	508	100
	J2-M	19.6	9.56	8.19	0.05	0.07	515	70
	J2-D	17.6	9.56	8.7	0.02	0.18	541	60
	J3-U	18.7	9.77	8.74	0.01	0.18	527	80
	J3-M	18.7	9.77	8.74	0.01	0.18	527	80
	J3-D	18.7	9.7	8.74	0.01	0.18	527	80
	J4-U	18.7	9.8	8.77	0.01	0.18	542	80
	J4-M	18.4	9.52	8.68	0.03	0.19	545	70
	J4-D	18.2	9.86	8.87	0.01	0.18	543	50
	J5-U	19.2	10.28	9.05	0.02	0.21	535	50
	J5-M	18.4	9.52	8.68	0.03	0.19	545	70
	J5-D	18.2	9.05	8.62	0.03	0.19	545	40
	J6-U	18.2	9.05	8.62	0.03	0.19	545	40
	J6-M	18.2	9.86	8.87	0.01	0.18	543	50
	J6-D	18.2	9.86	8.87	0.01	0.18	543	50
May	J1-U	24.4	10.14	8.75	0.02	0.15	384	70
	J1-M	25.8	10.35	8.45	0.02	0.14	395	50
	J1-D	25.8	10.35	8.46	0.02	0.15	383	50
	J2-U	23.9	10.04	8.47	0.02	0.15	388	50
	J2-M	23.9	10.04	8.47	0.02	0.15	388	50
	J2-D	23.9	10.1	8.4	0.02	0.15	395	50
	J3-U	23.5	8.38	8.5	0.02	0.15	409	50
	J3-M	25.7	8.38	8.47	0.01	0.15	373	60
	J3-D	25.5	10.34	8.46	0.02	0.15	382	40
	J4-U	23.6	8.7	8.62	0.01	0.15	392	40
	J4-M	23.6	8.7	8.62	0.01	0.15	392	40
	J4-D	25.9	10.31	8.46	0.02	0.15	374	50

Time	Site	TEMP (℃)	DO (mg/L)	pH	FV (M/s)	SAL	EC (μs/cm)	TRANSPARENCY (cm)
	J5-U	25.3	10.31	8.44	0.02	0.14	369	50
	J5-M	25.1	8.49	8.47	0.02	0.16	416	50
	J5-D	25.5	8.49	8.45	0.02	0.16	405	50
	J6-U	27.2	6.81	8.36	0.01	0.14	360	50
	J6-M	27.2	6.81	8.4	0.01	0.16	404	50
	J6-D	26.6	7.38	8.4	0.05	0.13	387	70
July	J1-U	31.6	10.35	9.33	0.14	0.13	372	40
	J1-M	31.6	9.35	9.33	0.10	0.13	372	40
	J1-D	31.6	10.35	9.33	0.07	0.13	372	40
	J2-U	31.9	10.04	9.28	0.15	0.12	380	50
	J2-M	31.9	10.04	9.28	0.08	0.12	380	50
	J2-D	31.9	10.04	9.28	0.02	0.12	380	50
	J3-U	32	10.34	9.24	0.05	0.14	337	50
	J3-M	32	10.34	9.24	0.05	0.14	337	50
	J3-D	32	10.34	9.24	0.01	0.14	337	50
	J4-U	31.7	10.31	9.11	0.20	0.15	413	60
	J4-M	31.7	10.31	9.11	0.12	0.15	413	60
	J4-D	31.7	10.31	9.11	0.05	0.15	413	60
	J5-U	26.9	8.7	8.7	0.18	0.14	400	60
	J5-M	26.9	8.7	8.7	0.11	0.14	400	60
	J5-D	26.9	8.7	8.7	0.07	0.14	400	60
	J6-U	26.9	8.7	8.7	0.18	0.14	400	60
	J6-M	29.5	7.38	8.45	0.17	0.15	363	70
	J6-D	29.5	7.38	8.45	0.04	0.15	363	70
November	J1-U	17.1	10.52	8.04	0.08	0.14	502	50
	J1-M	17.1	10.52	8.04	0.06	0.15	502	50
	J1-D	14.8	8.99	8.04	0.03	0.14	515	40
	J2-U	16.6	9.59	8.01	0.10	0.14	510	60
	J2-M	16	9.28	7.95	0.08	0.14	498	50
	J2-D	15.5	9.4	8.03	0.03	0.15	518	50
	J3-U	15	8.97	8.07	0.09	0.14	505	50

Time	Site	TEMP （℃）	DO （mg/L）	pH	FV （M/s）	SAL	EC （μs/cm）	TRANSPARENCY （cm）
	J3-M	15.1	9.07	7.99	0.05	0.14	503	40
	J3-D	15.9	9.68	8.03	0.02	0.15	509	60
	J4-U	12.2	9.9	8.06	0.05	0.14	530	40
	J4-M	12.7	9.51	8.05	0.03	0.14	521	50
	J4-D	12.2	9.33	8.04	0.05	0.14	513	40
	J5-U	15.5	8.99	8.01	0.06	0.14	710	60
	J5-M	12.3	9.6	8.05	0.04	0.13	519	60
	J5-D	12.3	9.17	8.04	0.02	0.14	537	50
	J6-U	12.8	8.99	8.05	0.07	0.14	506	40
	J6-M	12.8	8.99	8.05	0.07	0.14	506	40
	J6-D	12.8	8.99	8.05	0.07	0.14	506	40
